# Supplementary material for: Transient frontal spectral events from EEG predict antidepressant response to sertraline in depression
Source: medRxiv. 2026 Jan 27:2026.01.26.26344862. Preprint. [Version 1] doi: 10.64898/2026.01.26.26344862 (PMC12870669; doi:10.64898/2026.01.26.26344862)
Supplement: Supplement 1 [file NIHPP2026.01.26.26344862v1-supplement-1.pdf]

medRxiv preprint doi: https://doi.org/10.64898/2026.01.26.26344862; this version posted January 27, 2026. The copyright holder for this preprint (which was not certified by peer review) is the author/funder, who has granted medRxiv a license to display the preprint in perpetuity.
It is made available under a CC-BY 4.0 International license .

Supplementary Information

Determination of Factor-of-Median (FOM) threshold cutoff

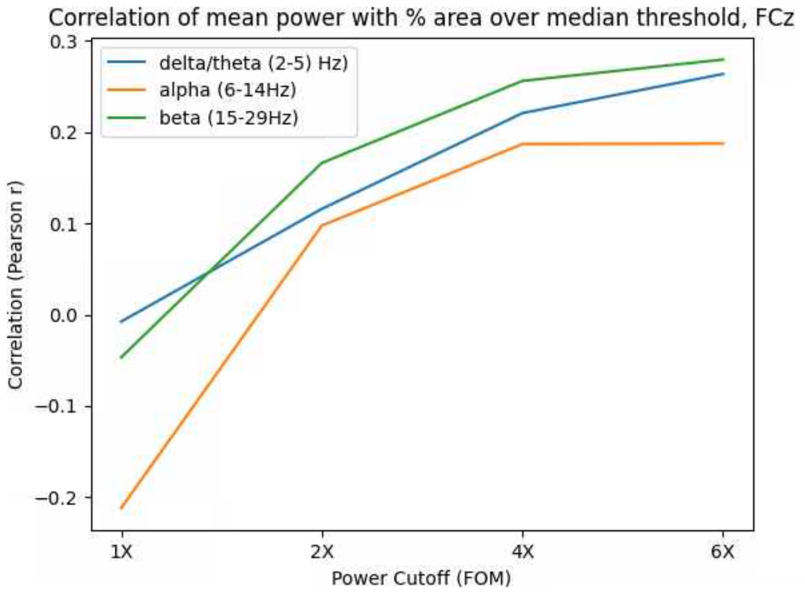

Supplementary Figure 1. Correlation between the power in the area above cutoff (that is, frequencies and time points that would be counted as transient events) in the frequency x time spectrogram and mean power in the frequency bands of interest. 6 x FOM had the highest correlation with mean power in all frequency bands, validating this choice of cutoff in the current study.

medRxiv preprint doi: https://doi.org/10.64898/2026.01.26.26344862; this version posted January 27, 2026. The copyright holder for this preprint (which was not certified by peer review) is the author/funder, who has granted medRxiv a license to display the preprint in perpetuity.
It is made available under a CC-BY 4.0 International license .

Model training, validation, and testing

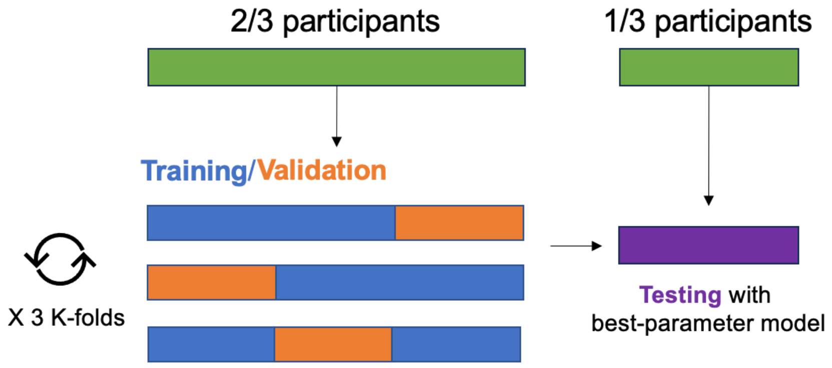

Supplementary Figure 2. Diagram of model training/validation and testing procedures. The cross-validated grid search and training/validation procedure was performed on 2/3 of participants using 3 K-folds for cross-validation. Testing was performed on the held-out 1/3 of participants using the resulting model with the best hyperparameters.

medRxiv preprint doi: https://doi.org/10.64898/2026.01.26.26344862; this version posted January 27, 2026. The copyright holder for this preprint (which was not certified by peer review) is the author/funder, who has granted medRxiv a license to display the preprint in perpetuity.
It is made available under a CC-BY 4.0 International license .

Range of SEF in Sertraline Group

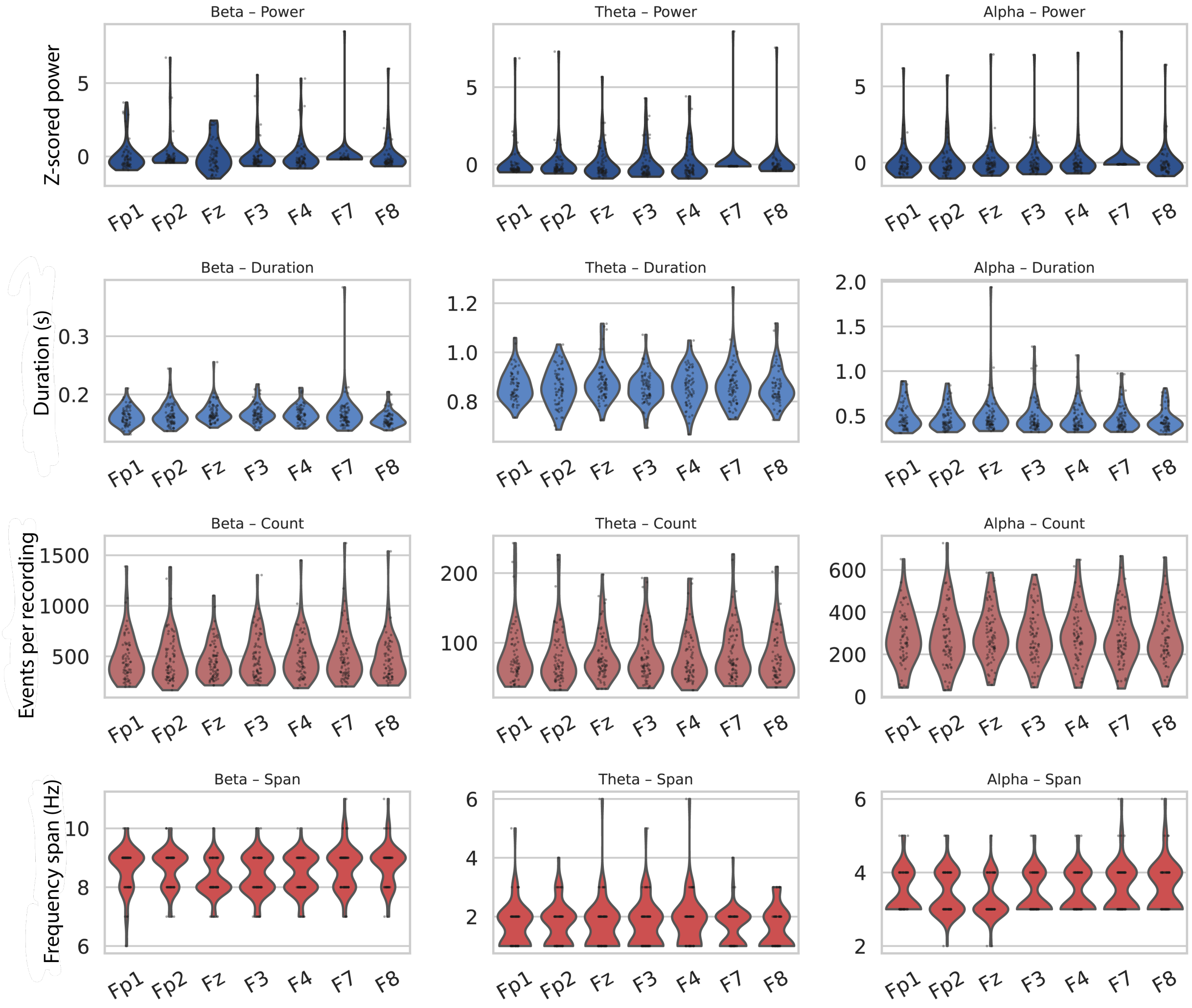

Supplementary Figure 3. Range of SEF at each channel and band for the subsample (N = 82) who received sertraline treatment.

medRxiv preprint doi: https://doi.org/10.64898/2026.01.26.26344862; this version posted January 27, 2026. The copyright holder for this preprint (which was not certified by peer review) is the author/funder, who has granted medRxiv a license to display the preprint in perpetuity.
It is made available under a CC-BY 4.0 International license .

Models cannot significantly classify binary sertraline treatment response/nonresponse with SEF only, APF, or SEF+APF

As described in the Methods and Results section, classification models predicting categorical (responder vs. non-responder) treatment outcomes from baseline SEF did not perform significantly above chance. For completeness and to compare performance against models presented in the literature, we also tested the performance of classification models constructed with Average Power Features only and with both SEF and APF. None of the classification models tested performed above chance at predicting sertraline responder status in the EMBARC dataset (see **Supplementary Table 1**).

Features included	*Model type*	*Predicted variable*	*Accuracy (F1): Training/Testing*	*P value: Training/Testing*
APF only[1]	KNN	Response (y/n)	.64	-
APF only[2]	SVM	Response (y/n)	.79	-
APF only[3]	RF	Response (y/n)	.88	-
SEF only	SVM	Response (y/n)	.54/.58	.27/.17
APF	SVM	Response (y/n)	.54/.35	.29/.70
APF+SEF	SVM	Response (y/n)	.49/.60	.53/.14
SEF only	KNN	Response (y/n)	.02/.42	.64/.45
APF	KNN	Response (y/n)	.60/.39	.09/.78
APF+SEF	KNN	Response (y/n)	.59/.38	.07/.56
SEF only	RF	Response (y/n)	.93/.94	.37/.22
APF	RF	Response (y/n)	.52/.61	.38/.13
APF+SEF	RF	Response (y/n)	.45/.22	.68/.95

Supplementary Table 1. Accuracy and significance of published models of binary classification models of antidepressant response and classification models tested in the current study. 1: Schwartzmann et al., 2023; 2: Zhdanov et al., 2020; 3: Jaworska et al., 2019.

medRxiv preprint doi: https://doi.org/10.64898/2026.01.26.26344862; this version posted January 27, 2026. The copyright holder for this preprint (which was not certified by peer review) is the author/funder, who has granted medRxiv a license to display the preprint in perpetuity.
It is made available under a CC-BY 4.0 International license .

Support Vector Regression does not significantly predict treatment response with the non-linear radial basis kernel with SEF, APF, or both.

To assess whether models that implement non-linear combinations of features instead (as opposed to linear combinations in our EN model) 1) significantly predict %change in HAM-D from SEF and/or 2) have performance improved by the inclusion of SEF, we constructed and tested the performance of Support Vector Regression (SVR) models using the radial basis kernel. SVR did not perform above chance at predicting %change HAM-D with APF, or a combination of both SEF and APF. However, SVR with SEF alone yielded a significantly predictive training-set model, though the testing-set model did not quite reach significance. This could indicate potential utility of non-linear combinations of SEF in predicting treatment response.

Features included	*Model type*	*Predicted variable*	*NRMSE: Training/Testing*	*P value: Training/Testing*
SEF only	SVR	%change HAM-D	-38.10/-34.44	.02/.06
APF	SVR	%change HAM-D	-40.20/-39.00	.35/.74
APF+SEF	SVR	%change HAM-D	-0.50/-0.52	.14/.72

Supplementary Table 2. Accuracy and significance of a non-linear model of %change HAM-D (i.e., support vector regression with the radial basis kernel) tested in the current study.

medRxiv preprint doi: https://doi.org/10.64898/2026.01.26.26344862; this version posted January 27, 2026. The copyright holder for this preprint (which was not certified by peer review) is the author/funder, who has granted medRxiv a license to display the preprint in perpetuity.
It is made available under a CC-BY 4.0 International license .

Sensitivity of model outcomes to factor-of-median SEF detection threshold

To test the effect of chosen factor-of-median detection threshold used to quantify spectral events in the SpectralEvents toolbox, we tested model performance when using SEF derived from events quantified at 4 x and 2 x factor-of-median power. We found that models including SEF quantified at these thresholds did not successfully generalize to predict above-chance in the validation stage.

Features included	*Model type*	*Predicted variable*	*NRMSE: Training/Testing*	*P value: Training/Testing*
SEF only (6xFOM)	EN	%change HAM-D	-37.34/-32.03	.005/.03
SEF only (4xFOM)	EN	%change HAM-D	-37.17/-35.68	.004/.13
SEF only (2xFOM)	EN	%change HAM-D	-36.76/-40.77	.002/.40

Supplementary Table 3. Accuracy and significance of elastic net models with SEF extracted using different factor-of-median detection thresholds.

medRxiv preprint doi: https://doi.org/10.64898/2026.01.26.26344862; this version posted January 27, 2026. The copyright holder for this preprint (which was not certified by peer review) is the author/funder, who has granted medRxiv a license to display the preprint in perpetuity.
It is made available under a CC-BY 4.0 International license .

Frontopolar beta duration does not differ between pre-treatment baseline and 1 week on sertraline.

While baseline Fp1 duration is predictive of post-treatment symptom changes after eight weeks of treatment, we did not see a significant change in beta duration one week into treatment ($t = -1.27$, $p = .22$), which is the only other timepoint at which EEG was collected in this study.

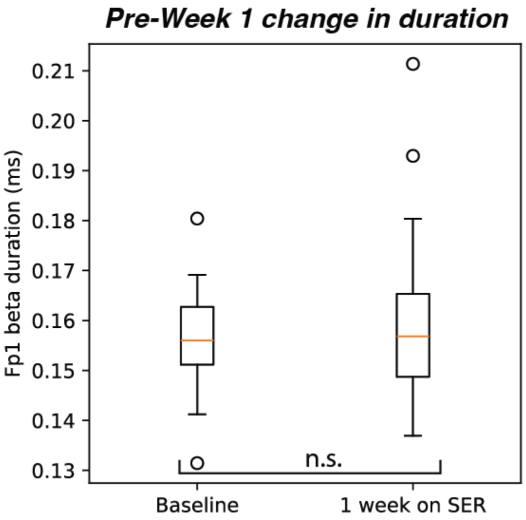

*Supplementary Figure 4. Fp1 beta duration does not significantly change during one week of sertraline treatment. * = p < .05; n.s. = not significant.*
